# Water Blooms—A Potential Threat to Male Reproduction: Clues From Aquatics and Rodents

**DOI:** 10.3389/fendo.2022.877292

**Published:** 2022-05-25

**Authors:** Shengdi Liu, Bin He, Hua Li

**Affiliations:** Department of Emergency Medicine, The First Affiliated Hospital of Nanjing Medical University, Nanjing, China

**Keywords:** microcystins, sperm, male reproductive system, testosterone, MC-LR

## Abstract

Toxic cyanobacteria blooms are a potential threat to global aquatic ecosystems and human health. Microcystin-leucine-arginine (MC-LR) is the most toxic variant of microcystins (MCs), and exposure to MCs can damage the male reproductive system. Two electronic databases were searched for controlled studies of rodents and fishes published before September 2020. Effect sizes were calculated for eight main reproductive parameters, including sperm count, sperm motility, sperm morphology, serum testosterone, testis weight, serum follicle stimulating hormone (FSH), serum luteinising hormone (LH) and serum estradiol. Nine meta-analyses of individual parameters were conducted using R version 4.0.2. Fifteen studies were included in the meta-analysis. In the studies of rodents, exposure to MC-LR by intraperitoneal injection or intragastric administration yielded statistically significant effects on sperm count (standardised mean difference (SMD) = -1.7426 (95% CI: -2.2098 to -1.2754)), abnormal sperm rate (SMD = 1.6714 (95% CI: 0.9702 to 2.3726)), sper5% CI: -3.9811 to -1.7834)), testis weight (SMD = -2.8822 (95% CI: -3.9811 to -1.7834)) and serum FSH (SMD = 0.4707 (95% CI: 0.0659 to 0.8756) changes in serum testosterone (SMD = 0.5521 (95% CI: 0.1652; 0.9391)) and estradiol (SMD = 0.6398 (95% CI: 0.1896 to 1.0900)) concentrations are considered to be statistically significant. Dose–response analysis reflected the dynamic changes of male reproductive function caused by MC. Short-term exposure to MC-LR can affect the function of the male reproductive system in rodents and fish. Elevated dosage or extended exposure time may worsen the damage. Human-related research on MC-LR exposure is very necessary to protect health and the water environment.

## 1 Introduction

With the acceleration of urban industrialisation around the world, industrial and domestic wastewater, which contains a large number of pollutants, is being discharged into rivers and lakes, resulting in increasingly serious eutrophication, which will lead to increased incidence of cyanobacteria blooms (1). Microcystins (MCs) are a kind of toxoid produced by freshwater cyanobacteria, including *Microcystis*, *Aphanizomenon*, *Nostoc* and *Anabaena*. Recently, with the frequent outbreaks of cyanobacteria blooms, more and more lakes are facing MC pollution (2). Amongst the over 250 known microcyanobacteria toxins, microcystin-leucine-arginine (MC-LR) has the broadest distribution and strongest toxicity. The World Health Organization limits the MC-LR concentration in drinking water to 1 µg/L (3). In China, the concentration of dissolved MC in Taihu Lake reached the highest level of 35.8 ug/L in October 2008, which greatly affected the living environment of fish and mammals (4). MC-LR can affect many organs, such as the thyroid, brain, liver, heart, intestinal tract, kidney and reproductive organs. MC-LR primarily accumulates in the liver, followed by the gonad (5).

At present, about 60,000 people suffer from food poisoning caused by cyanobacteria blooms every year (6). In China, as lake water is an important source of drinking water, its source condition directly affects the quality and safety of millions of people. Every year, a great deal of manpower, material and financial resources are spent to control cyanobacteria bloom (7). Since 2007, the cost of cleaning up Taihu Lake has exceeded 16 billion US dollars (8). Although a large amount of money has been spent on dealing with cyanobacteria blooms in Taihu Lake, the cyanobacteria bloom is still inevitable when the temperature increases every spring and summer. Compared with 2007–2018, the outbreak of cyanobacteria bloom in Taihu Lake occurred 29 days earlier in 2019 (9). Millions of people drink water from Taihu Lake, which is polluted by cyanobacteria blooms every year and may be hazardous to human organs (10).

The quality and motility of male sperms have been declining in the past 50 years, which are affected by many factors, including tobacco and alcohol use, irregular daily routine and environmental toxicants (11). Amongst them, various environmental toxins, such as dibutyl phthalate, diethylhexyl phthalate and pyrethrins, have been proved to damage male spermatozoa (12). Because these endocrine disruptors have strong estrogenic effects, they can cause great damage to the male reproductive system; moreover, they can not only damage the spermatogenesis of males exposed to these chemicals directly but also influence their male offsprings (13).

Several studies have found that MCs exhibit reproductive toxicity to male fish, mammals and frogs. After exposure to MCs for 14 days, the testis of mouse atrophied. Histological observation showed that MCs could damage the testicular structure compared with the control group (5). Further studies have shown that MC-LR can induce oxidative stress in the testis of mature rats (14). MCs can interfere with the endocrine system and induce abnormal reproductive activity in male fish, rats and frogs. Jia reported that exposure to low dosage of MC-LR damaged the reproductive system of male frogs, and oxidative damage was observed (6). Chen et al. found testicular inflammation and macrophage and lymphocyte infiltration in mouse testis after MC-LR administration (15). Another study showed that exposure to MC-LR during pregnancy or lactation can damage the reproductive function of male offspring. Compared with the control group, offspring exposed to MCs showed decreased testicular weight and disordered arrangement of spermatogenic cells with increased apoptosis rate (16).

In recent years, an increasing number of studies on mammals and fish have begun to evaluate the effects of MCs on the male reproductive system. Therefore, the purpose of this meta-analysis is to identify and evaluate the epidemiological evidence associated with exposure to MCs and damage to the male reproductive system of fish and rodents. Microcystins are highly enriched in the water environment, so fish studies are of great significance to understand the latest status of microcystins. Besides, water is necessary for human. mouse, as a kind of mammals, are closely related to human, which may suggest the reproductive damage on human.

## 2 Materials and Methods

### 2.1 Search Strategy

This meta-analysis was performed in accordance with the guidelines of the Preferred Reporting Items for Systematic Reviews and Meta-analyses. On September 1, 2020, literature retrieval was performed independently by two researchers (Shengdi Liu and Bin He) in PubMed and Embase. Our search strategy is as follows: (sperm count) OR (sperm concentration)) OR (sperm morphology)) OR (sperm motility)) OR (sperm energy metabolism)) OR (sperm viability)) OR (sperm fertilization capacity)) OR (sperm capacitation reaction)) OR (sperm acrosome reaction)) OR (follicle stimulating hormone)) OR (luteinizing hormone)) OR (testosterone, estrogen)) OR (testicular size)) OR (testicular weight)) OR (sexual drive) AND [(microcystin*) OR (MC-LR)].

### 2.2 Study Selection and Eligibility Criteria

Our meta-analysis included rodent and fish studies. The titles and abstracts of the retrieved articles were independently screened (Shengdi Liu and Bin He).

The inclusion criteria were as follows: (1) MC is the only exposed pollutant. (2) The article should be published between January 1990 and June 2020. (3) The study should report reproductive parameters such as sperm count, sperm motility, sperm morphology or serum testosterone concentration. (4) The study should include appropriate data, which is suitable for conducting a meta-analysis.

The exclusion criteria were as follows: (1) *in vivo* experimental studies, (2) ecological studies on the exposure at the population level rather than at the individual level and (3) studies without valuable data on phenotypic changes for analysis.

### 2.3 Data Extraction and Quality Assessment

To minimise deviation and improve reliability, three researchers (Shengdi Liu, Hua Li and Bin He) extracted the data independently and resolved their differences through discussion. In addition to data on the average and standard deviation of relative parameters of sperm and testis with and without exposure to MCs, the following data were extracted: first author, dates on which the country was published, publication year, species of the study object, exposure period and dose. Engauge Digitizer version 5.0 (https://sourceforge.net/projects/digitizer/) was used to extract information from figures. After extracting the data, a researcher checked for differences in the data to minimise the possibility of error. Risk of bias was assessed by two researchers (Shengdi Liu and Bin He) focussing on nine domains, including randomisation, assignment concealment, confusion (design/analysis), accidental exposure, same experimental conditions, compliance with scheme, blindness of researchers during the study, lack of outcome data and evaluation of confusing variables.

### 2.4 Data Synthesis and Analysis

All statistical analyses were performed in R version 4.0.2 (R Statistical Computing Foundation, Vienna, Austria; http://www.R-project.org). The pooled standardised mean differences (SMDs) between the control group and the exposure group were calculated to determine the effect size. The fixed effect model and random effect model (REMS) were fitted to evaluate the model type that is most suitable for the data. ‘Car’ packages were used to evaluate the dose–response relationship. Q test and I^2^ statistics were used to evaluate the heterogeneity. Statistical significance was considered at P < 0.05. Subgroup meta-analysis was conducted to investigate the effects of different exposure doses and times. We used a funnel chart to directly compare 10 or more studies to assess publication bias. Sensitivity analysis was conducted to evaluate the effect of individual studies on the estimation of comprehensive effects, and we analysed the impact of each study on pooled SMD by deleting them.

## 3 Results

### 3.1 Literature Selection and Characteristics of the Included Studies

**Figure 1:**
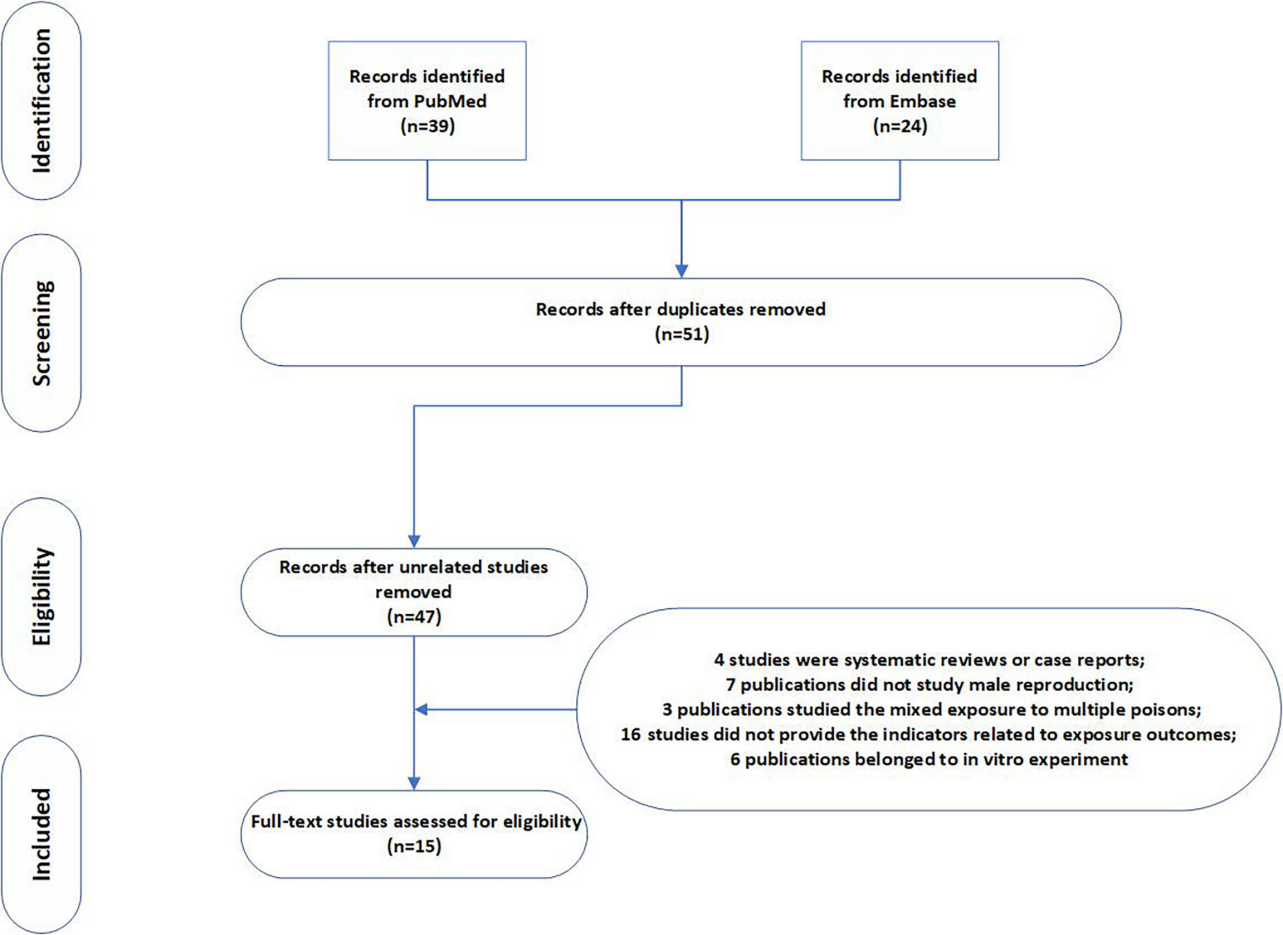
Flow diagram of literature search strategy for the meta-analysis.

We combined medical subject headings and generic terms for the exposures and outcomes. A total of 39 and 24 results were obtained from PubMed and Embase database, respectively. After deleting repetitive literature, we obtained a total of 51 related articles. We evaluated the full text of the 51 articles. Amongst them, 36 articles did not meet the inclusion criteria, 4 articles were systematic reviews or case reports, 7 articles did not study male reproduction, 3 articles studied mixed exposure to multiple pollutants, 16 articles did not provide the indicators related to exposure outcomes, and 6 articles belonged to *in vitro* experiments. The selection process is shown in the flow chart. After screening, a total of 15 studies were included in the meta-analysis, including nine rodent studies (5, 14, 17–23) and six fish studies. Seven indicators were measured in our study (**Figure 1**). **Table 1** shows the characteristics of the included studies by year of publication and first author, including the results of studies. The articles were published from 2006 to 2019, and more than 90% of the articles were published in the last 10 years. Studies were mostly conducted in China, and the reagent used in all experiments was MC-LR. The exposure time varied from 1 day to 90 days, and the exposure dosage ranged from 1 µg/L to 30 µg/L.

**Table 1:** The characteristics of the included studies by year of publication and first author, including the results of studies.

Author	Year	Experimental object	country	exposure time	exposure mode	Research content
Yabing Chen	2017	mice	USA	3 monthes	Intraperitoneal injection	sperm count,Sperm abnormal percentage
Yu Chen	2011	mice	China	3 monthes	Intragastric instillation	sperm count,Sperm abnormal percentage,sperm motility, FSH, serum Testosterone,LH
Xin Xiang	2006	mice	China	14 days	Intraperitoneal injection	sperm count,Sperm abnormal percentage,testis weight, sperm motility
Yan Li	2008	rat	China	28 days	Intraperitoneal injection	sperm count,Sperm abnormal percentage,sperm motility, FSH, serum Testosterone,LH
Xueting Wang	2016	rat	China	14 days	Intraperitoneal injection	serum Testosterone
Xueting Wang	2012	mice	China	1 day	Intraperitoneal injection	FSH, serum Testosterone,LH
Xiaolu Xiong	2014	mice	China	1 day	Intraperitoneal injection	FSH, serum Testosterone,LH,sperm count
Ling Zhang	2018	mice	China	14 days	Intraperitoneal injection	sperm count,Sperm abnormal percentage,sperm motility, testis weight
Yuan Zhou	2013	rat	China	7 days	Intraperitoneal injection	testis weight
Jiazhang Chen	2017	Nile tilapia	China	28 days	immersion	Serum estradiol, serum Testosterone
Jie Hou	2018	zebrafish	China	30 days	immersion	Serum estradiol, serum Testosterone
Wang Lin	2018	Zebrafish	China	30 days	immersion	Serum estradiol, serum Testosterone
Wanjing Liu	2016	zebrafish	China	30 days	immersion	Serum estradiol, serum Testosterone
Qin Qiao	2013	zebrafish	China	30 days	immersion	Serum estradiol, serum Testosterone
Yujing Su	2016	zebrafish	China	90 days	immersion	Serum estradiol, serum Testosterone

### 3.2 Male Reproductive Toxicity of MCs in Rats and Mouse

#### 3.2.1 Sperm Count

Amongst the included literature, six studies reported 37 results of sperm count. The pooled SMD by REMS was -1.7426 (95% CI: -2.2098 to -1.2754). The results were statistically significant (P < 0.0001) (**Supplementary Figure 1A**) in almost all studies. The dose–response diagram showed that low-dose and high-dose MC exposure led to a decreased sperm count, and even a single intragastric administration caused decreased sperm count in mouse or rats (**Supplementary Figure 4A**). This effect did not change with the species of rodent and the route of administration. The funnel chart showed a certain publication bias (**Supplementary Figure 2A**), and the sensitivity analysis showed that deletion of any estimates did not affect the results (**Supplementary Figure 3A**).

#### 3.2.2 Abnormal Sperm Rate

Five studies focused on the effect of MCs on abnormal sperm rate, providing 18 results. The pooled SMD by REMS was 1.6714 (95% CI: 0.9702 to 2.3726). The abnormal sperm rate in mouse and rats treated with *Microcystis* was significantly increased with statistical significance (P < 0.0001) (**Supplementary Figure 1B**). Moreover, the abnormal sperm rate was positively correlated with the time and dose as demonstrated in the dose–response diagram (**Supplementary Figure 4B**). The results are consistent. All studies found that MCs can increase the abnormal sperm rate. The funnel chart showed that the results have publication bias (**Supplementary Figure 2B**), and sensitivity analysis showed that the deletion of any study did not change the SMD (**Supplementary Figure 3B**).

#### 3.2.3 Sperm Motility

The motility of mouse spermatozoa decreased significantly after exposure to MCs. Only three articles provided 12 results, and the pooled SMD by REMS was -2.8822 (95% CI: -3.9811 to -1.7834). The results are statistically significant (P < 0.0001) (**Supplementary Figure 1C**). The sperm motility was seriously affected by MC exposure in a time- and dose-dependent manner. In addition, the damage effect seems to be related only to the time and dose of exposure, but not to the mode of exposure (**Supplementary Figure 4C**). The funnel chart indicated that the results have publication bias (**Supplementary Figure 2C**), and sensitivity analysis showed that the deletion of any study did not change the SMD (**Supplementary Figure 3C**).

#### 3.2.4 Testis Weight

Compared with the control group, the testis weight of mouse exposed to MCs was decreased. Moreover, with the increase of exposure time and dose, the testis weight decreased significantly. The pooled SMD by REMS was -2.8822 (95% CI: -3.9811 to -1.7834). We speculate that MCs may hinder testicular development, and the results are statistically significant (P < 0.0001) (**Supplementary Figure 1D**). The results suggest that the testis weight of rats and mouse decreased gradually with the increase of exposure time and dose, which can be observed in the dose–response diagram (**Supplementary Figure 4D**). Our funnel chart showed a certain publication bias in the meta-analysis (**Supplementary Figure 2D**), and sensitivity analysis showed that removing any of the studies did not change the pooled SMD (**Supplementary Figure 3D**).

#### 3.2.5 Serum Testosterone

Five studies provided 42 results showing decreased testis weight in rats and mouse after exposure to MCs. The pooled SMD by REMS was -0.1407 (95% CI: -0.6823 to 0.4010). The results were not statistically significant (P = 0.6108) (**Supplementary Figure 1E**). Interestingly, we found that in all the experimental groups, the serum testosterone levels in rats and mouse tended to increase when exposed with fewer doses and in a short time. However, long-term and high-dose MC exposure led to a decrease in serum testosterone levels (**Supplementary Figure 4E**). We conducted a subgroup analysis of the results. A dose less than 10 µg/L and exposure time less than two weeks were placed into one group for analysis, and the rest of the experiments were placed in another subgroup for analysis. The pooled SMD of the former was 1.1709 (95% CI: 0.4931 to 1.8487), whilst that of the latter was -1.4958 (95% CI: -2.1637; -0.8279). Both results were statistically significant (P < 0.01). The funnel chart showed that the meta-analysis had no published bias (**Supplementary Figure 2E**), and the sensitivity analysis showed that the removal of any study did not have a significant impact on the pooled SMD (**Supplementary Figure 3E**).

#### 3.2.6 Serum Follicle Stimulating Hormone (FSH) and Luteinising Hormone (LH)

The effect of MC exposure on FSH is not very significant, but the overall trend is rising. Low-dose exposure may increase FSH content slightly, whereas high-dose exposure may decrease FSH. This trend is also related to exposure time (**Supplementary Figure 4F**). The pooled SMD by REMS was 0.4707 (95% CI: 0.0659 to 0.8756), and the results were statistically significant (P = 0.0227) (**Supplementary Figure 1F**). A total of 41 data based on the changes of LH after exposure to MCs. The pooled SMD by REMS was -0.1417 (95% CI: -0.6958 to 0.4123), but the results were not statistically significant (P = 0.6161) (**Supplementary Figure 1G**). The funnel chart showed that the former has publication bias (**Supplementary Figure 2F**), whereas the latter has no publication bias (**Supplementary Figure 2G**). Sensitivity analysis showed that removing any of the studies did not change the overall SMD (**Supplementary Figures 3F, G**).

### 3.3 Male Reproductive Toxicity of MCs in Fishes

#### 3.3.1 Serum Testosterone

Long-term or short-term exposure to MCs can also interfere with fish testosterone levels, different from the above-mentioned studies of rats or mouse. In addition to the research by Wang Lin et al., the results demonstrated that testosterone levels in male fish exposed to MCs were increased. The pooled SMD by REMS was 0.5521 (95% CI: 0.1652; 0.9391), and the results were statistically significant (P = 0.0052) (**Supplementary Figure 1I**). Sensitivity analysis showed that removing the study of Wang Lin et al. reduced the P-value to 0.0003 and heterogeneity to 64.5% (**Supplementary Figure 3I**). The funnel chart showed publication bias (**Supplementary Figure 2I**).

#### 3.3.2 Serum Estradiol

In all included fish studies, exposure to MCs caused estradiol hormone disorders. The pooled SMD by REMS was 0.6398 (95% CI: 0.1896 to 1.0900), and the results were statistically significant (P = 0.0053) (**Supplementary Figure 1H**). The changes in serum estradiol levels were correlated with the exposure time and dose. The funnel chart showed that the meta-analysis has no publication bias (**Supplementary Figure 2H**), and the sensitivity analysis showed that the removal of any study did not have a significant impact on the pooled SMD (**Supplementary Figure 3H**).

### 3.4 Subgroup Analysis of the Toxicity of MCs to the Reproductive System

The risk of bias in the study was evaluated by subgroup analysis according to the exposure dose and time (**Supplementary Figures 5, 6**). In most subgroup analyses, we found statistically significant results consistent with the pooled analysis. However, in the analysis of serum testosterone, FSH and LH, the results of each subgroup were not consistent with the original results. In general, a deviation was observed in the combined effect caused by low and high doses of MCs. In the analysis of these subgroups, the value of I^2^ decreased, which indicated that the exposure time and dose affected the effect of MC exposure.

## 4 Discussion

In the past decade, environmental problems, which have attracted worldwide attention, are particularly serious in developing countries. Water bloom is one of the most serious environmental issues (1). Cyanobacteria blooms have caused the death of a large number of fishes and lead to extremely serious damage to the marine ecosystem (2). Although the Chinese government has been controlling cyanobacteria blooms in Taihu Lake for a long time, they are still rampant, which have drawn the attention of the World Health Organization (24). Although we are not directly exposed to MCs, they can enter the human body through the consumption of aquatic animals, including fish, frogs, plankton or aquatic plants, and are finally distributed in various organs of the body (25).

To our knowledge, this is the first meta-analysis that focuses on the negative effects of MC-LR on the male reproductive system. In total, nine individual meta-analyses in our systematic review were undertaken to calculate the pooled SMD of MC exposure. Amongst them, seven meta-analyses reached statistical significance (P < 0.05), and four meta-analyses showed significant positive associations. According to the data of rodent studies, sperm count (SMD = -1.7426 (95% CI: -2.2098 to -1.2754)), sperm motility (SMD = -2.8822 (95% CI: -3.9811 to -1.7834)) abnormal sperm rate (SMD = 1.6714 (95% CI: 0.9702 to 2.3726)) and serum FSH (SMD = 0.4707 (95% CI: 0.0659 to 0.8756)) demonstrated significant positive associations. According to the data of fish studies, significant positive associations were found in serum testosterone (SMD = 0.5521 (95% CI: 0.1652; 0.9391)) and serum estradiol (SMD = 0.6398 (95% CI: 0.1896 to 1.0900)). In the two remaining meta-analyses, although no significant correlation was found, serum testosterone and serum LH were still affected by MCs.

Jia et al. found a large amount of MC-LR accumulation in the testes of *Rana nigromaculata* by immunofluorescence staining. They found that the down-regulation of Hsd17b3 gene expression led to the inhibition of T synthesis, and that the up-regulation of CYP19A1 gene expression directly stimulated the transformation of testosterone to estradiol (7). In another study, the endocrine function of the male rana nigromaculata was affected after exposure to 1 µg/LMC in fresh water (16). MCs can affect the neurological function, sperm count, sperm motility, sperm viability, sperm progressive motility and sperm viability of rats. Histological examination showed that the testis atrophied and the structure of seminiferous tubules was damaged in the MC group (18). The main function of Leydig cells is to produce androgen, which is necessary for spermatogenesis, maturation and the maintenance of libido (20). A decrease in testosterone may reflect damage of interstitial cells caused by MC-LR exposure. As noted above, MCs likely pass through the blood–testis barrier and affect the spermatogenesis of the testis and the production of testosterone, resulting in the weakening of libido in male rats.

At present, the mechanism by which MC-LR affects the male reproductive system is unclear. Fish studies have found that MC-LR may delay gonadal maturation by interfering with the growth hormone/insulin-like growth factor system. Moreover, the gonadal development of males was found to be more easily affected by MC-LR than that of females (26). However, Qin Qiao et al. found that female zebrafish are more vulnerable to MC-LR exposure than males (27). In mouse studies, MC-LR can induce immune response of Sertoli cells, germ cells and interstitial cells by activating phosphatidylinositol 3-kinase/AKT/nuclear factor kappa B, thus producing proinflammatory cytokines and chemokines and damaging the function of the main testicular cells, which is crucial to the spermatogenic function and testosterone level of the testis (5).

This study has several limitations. The relationship between MC exposure and male reproductive system damage was affected by many factors, which may be the cause of heterogeneity, and the exposure time and dose may be the source of heterogeneity. In most teleost fish, the dominant male hormone should be 11-keto-testosterone (28). In addition, 11-keto-testosterone is found in higher levels in the plasma of males than in females, whereas this is usually not the case for testosterone (29). 11-keto-testosterone is generally more effective than testosterone in stimulating secondary sexual characters, reproductive behavior and spermatogenesis (30). However, studies that are involved in the meta-analysis did not explore the 11-keto-testosterone concentration of fishes with MCs exposure. We hope researches could focused more on the role of 11-keto-testosterone when exploring the damage on fish reproductive system. Despite these limitations, our findings have practical implications. We evaluated the effects of MC exposure on the reproductive system of mouse and fish. In addition, we conducted several subgroup analyses to explore the effects of several key factors on the study. Furthermore, the dose–response relationship curve can be used to intuitively observe the relationship between short-term and long-term exposure, as well as between low-dose and high-dose exposure.

## 5 Conclusion

The evidence provided by this meta-analysis suggested that exposure to MC-LR affects the male reproductive system, including the sperm, testis and testosterone. Moreover, the exposure time and dose affect the relationship between MC-LR exposure and parameters related to the male reproductive system. Further research should focus on the effects of long-term low-dose exposure and the effects of MC-LR on humans. In addition, it is also necessary to further explore the mechanism of MC-LR toxic effect on the male reproductive system.

## Data Availability Statement

The raw data supporting the conclusions of this article will be made available by the authors, without undue reservation.

## Authors Contributions

SL: Conceptualization, Methodology, Investigation, Writing - original draft preparation. BH: Data curation, Formal analysis, Writing - review and editing. HL: Supervision, Resources, Writing - review and editing. All authors contributed to the article and approved the submitted version.

## Conflict of Interest

The authors declare that the research was conducted in the absence of any commercial or financial relationships that could be construed as a potential conflict of interest.

## Publisher’s Note

All claims expressed in this article are solely those of the authors and do not necessarily represent those of their affiliated organizations, or those of the publisher, the editors and the reviewers. Any product that may be evaluated in this article, or claim that may be made by its manufacturer, is not guaranteed or endorsed by the publisher.
