# Supplementary figures and images for: Water Blooms—A Potential Threat to Male Reproduction: Clues From Aquatics and Rodents
Source: Front Endocrinol (Lausanne). 2022 May 25;13:877292. doi: 10.3389/fendo.2022.877292 (PMC9174978; doi:10.3389/fendo.2022.877292)

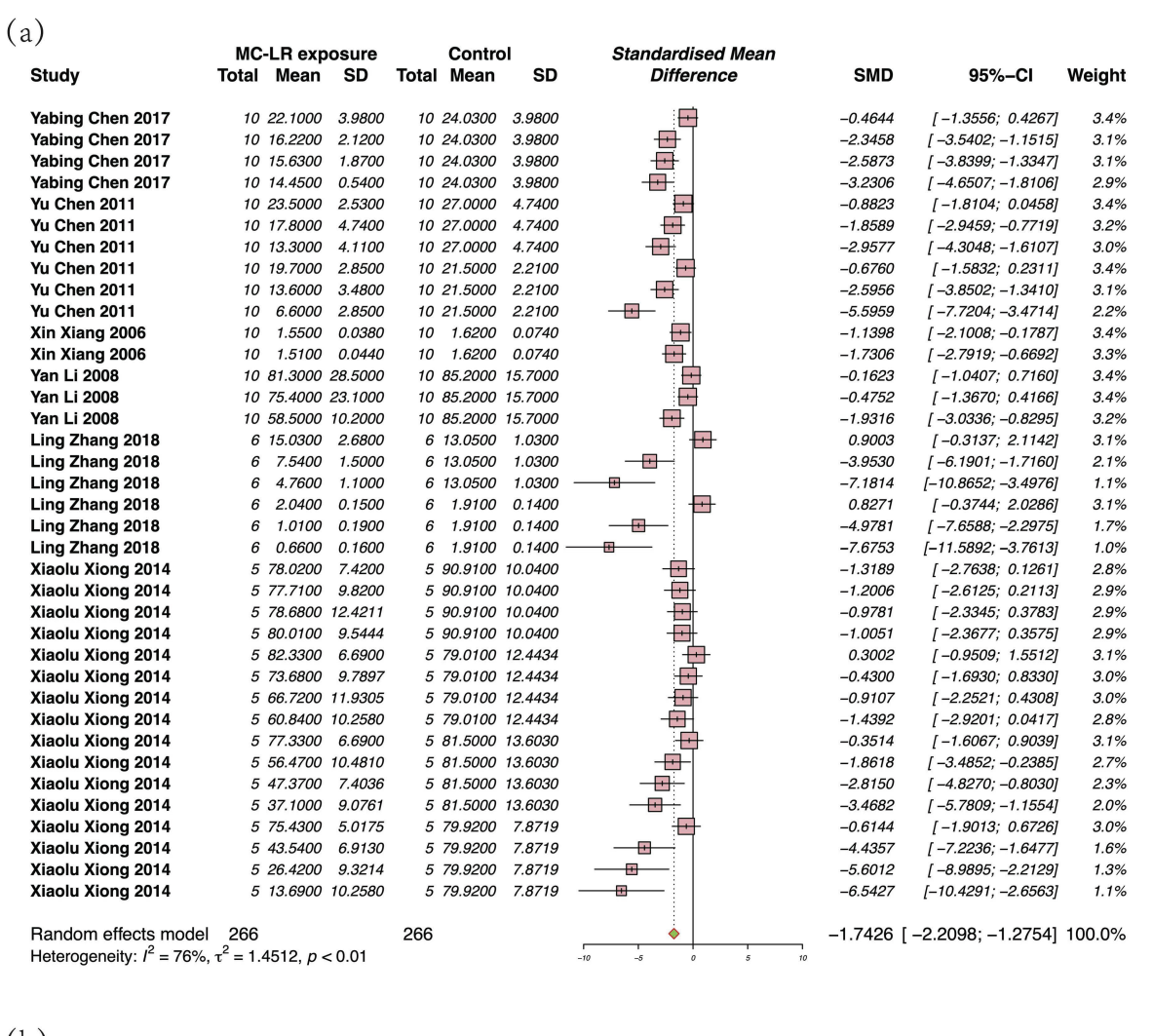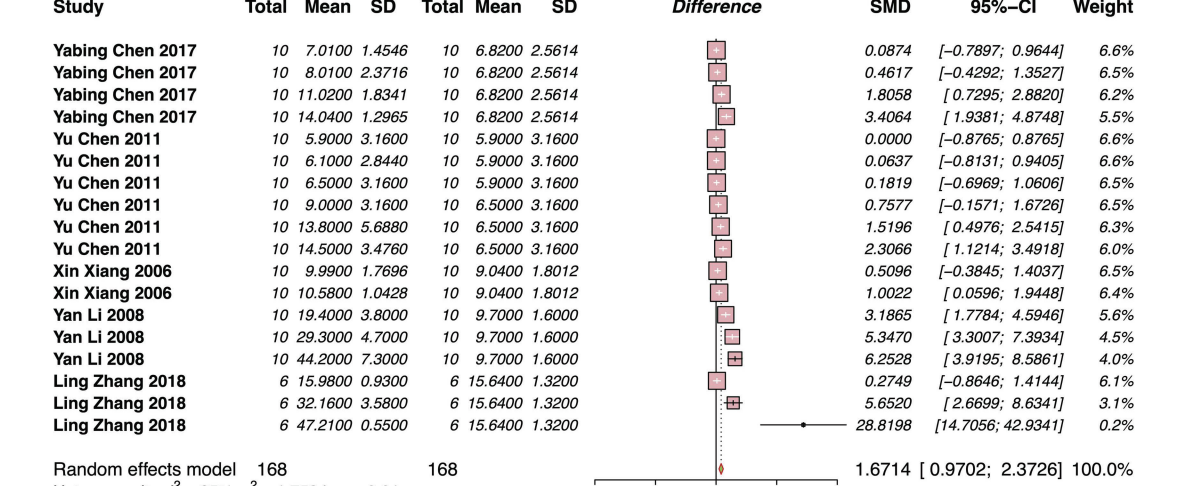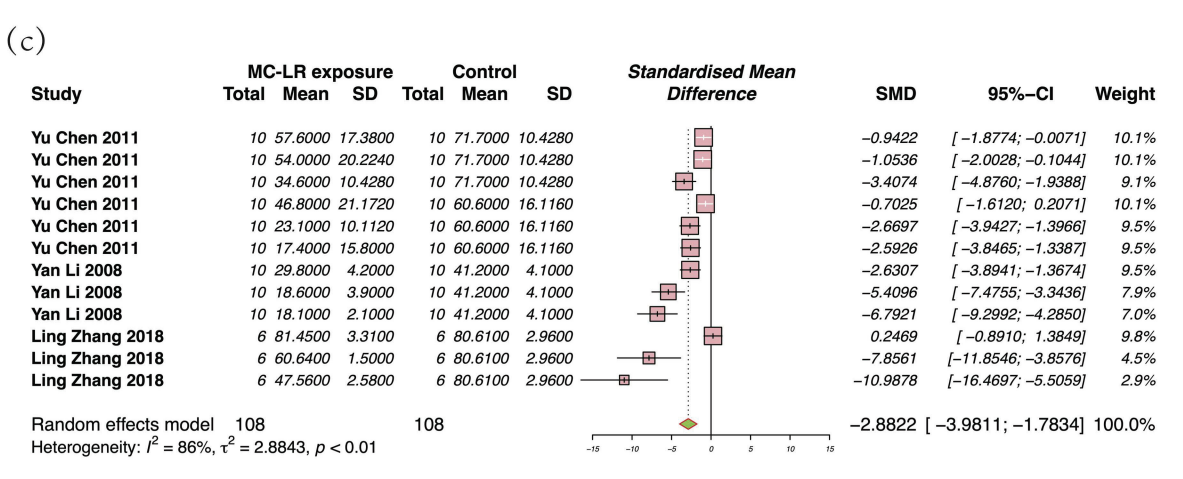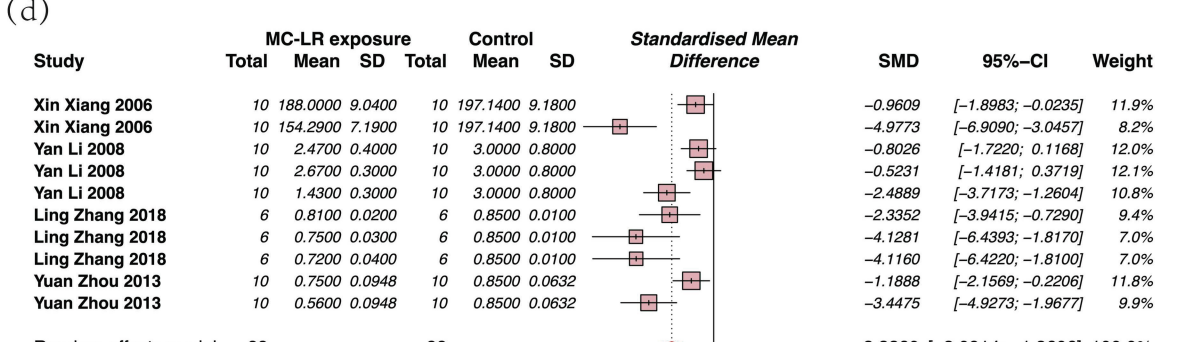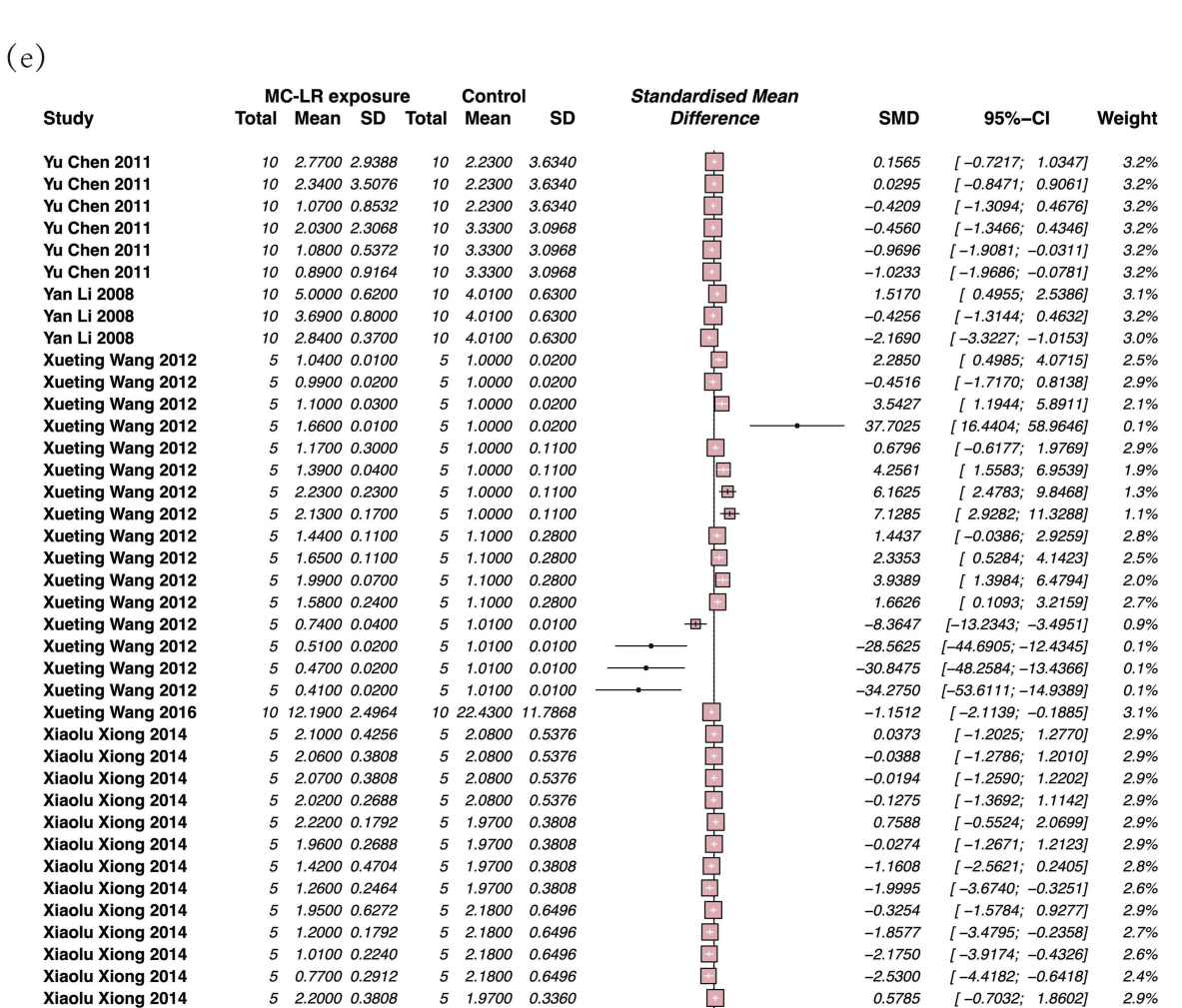

Supplement: Supplementary file 1 [file DataSheet_1.zip › Supplementary Figure 1.pdf]

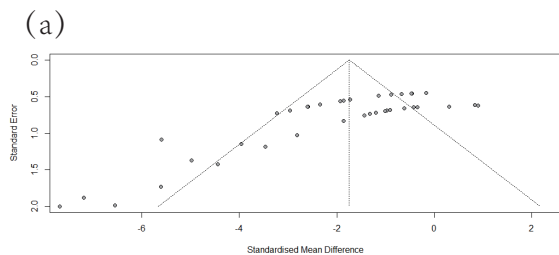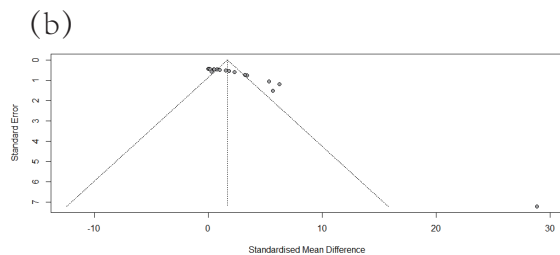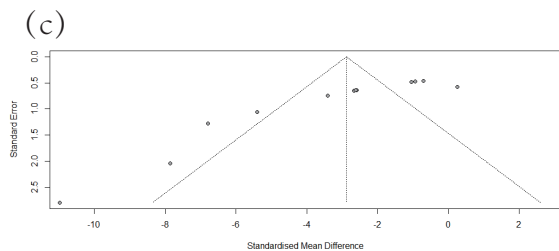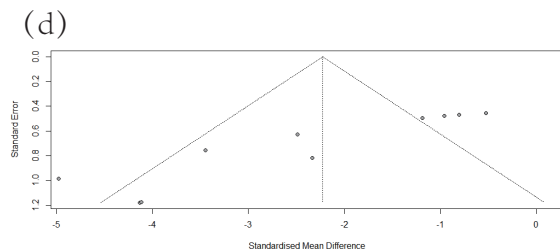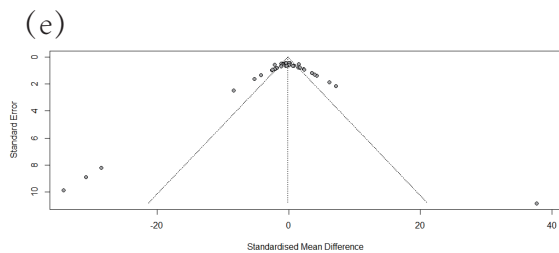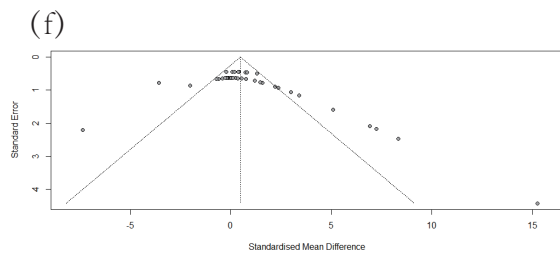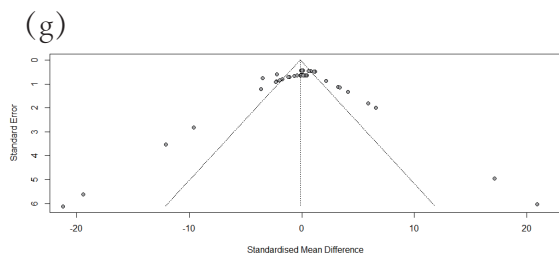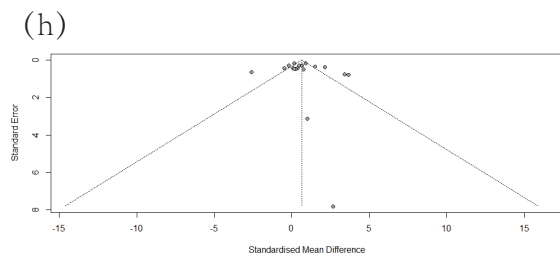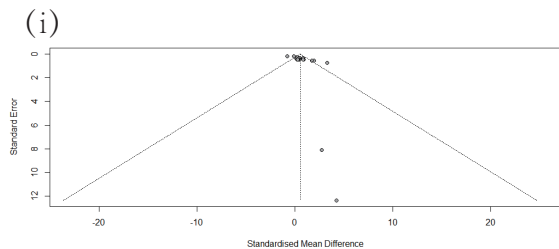

Supplement: Supplementary file 1 [file DataSheet_1.zip › Supplementary Figure 2.pdf]

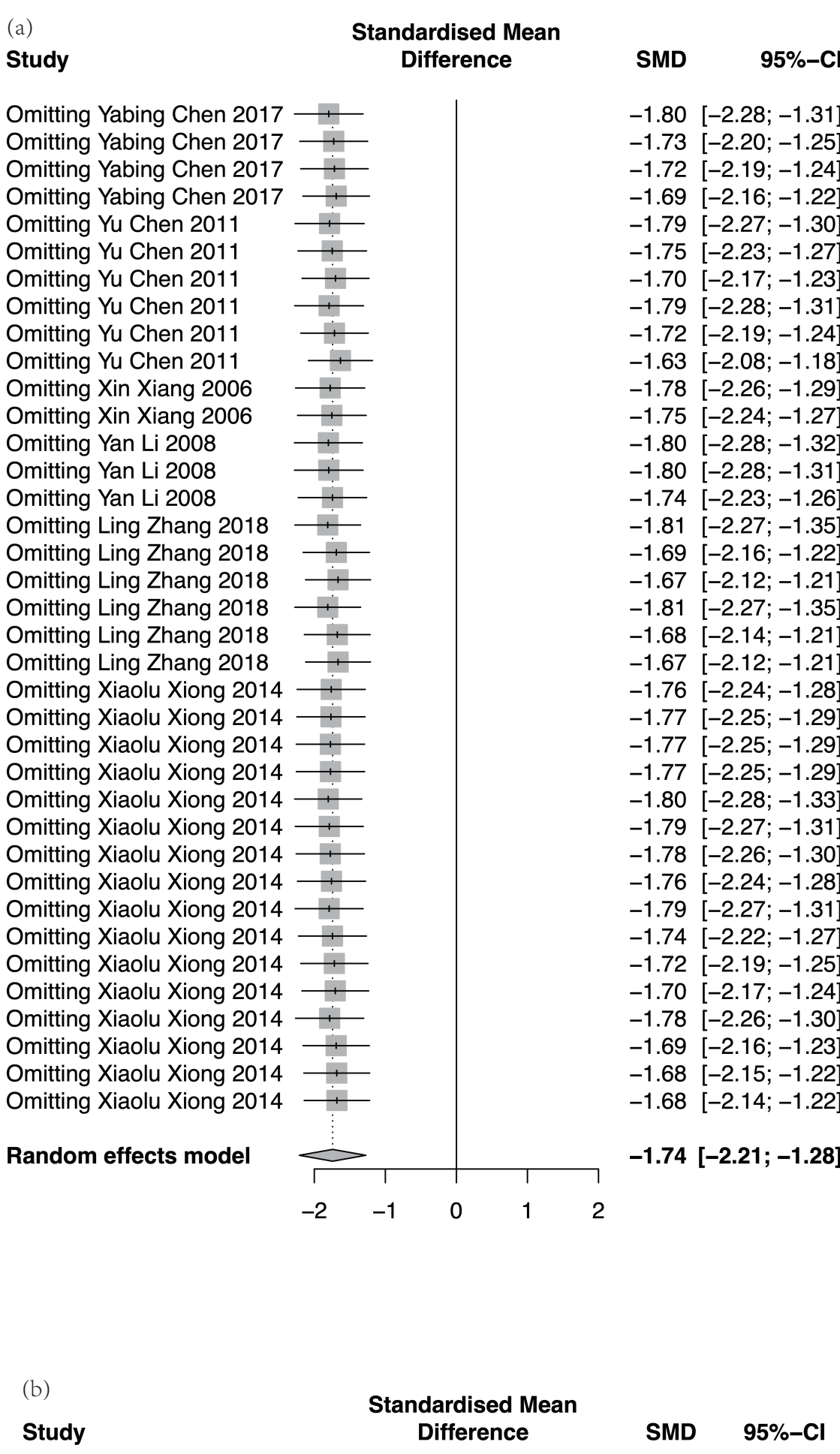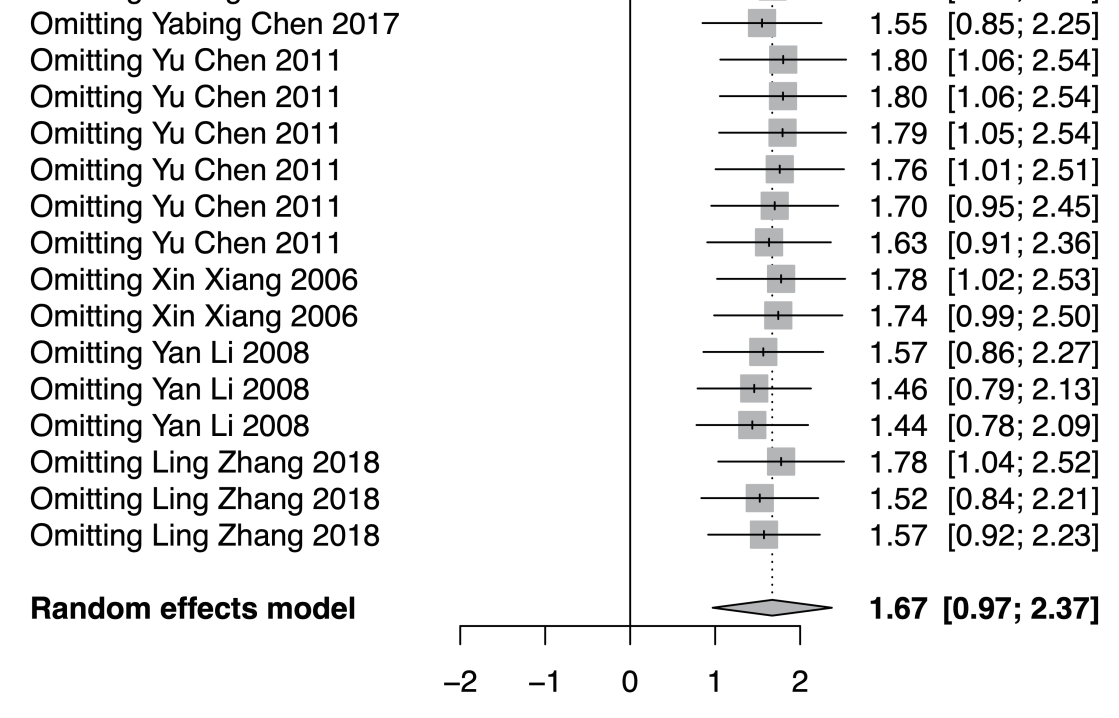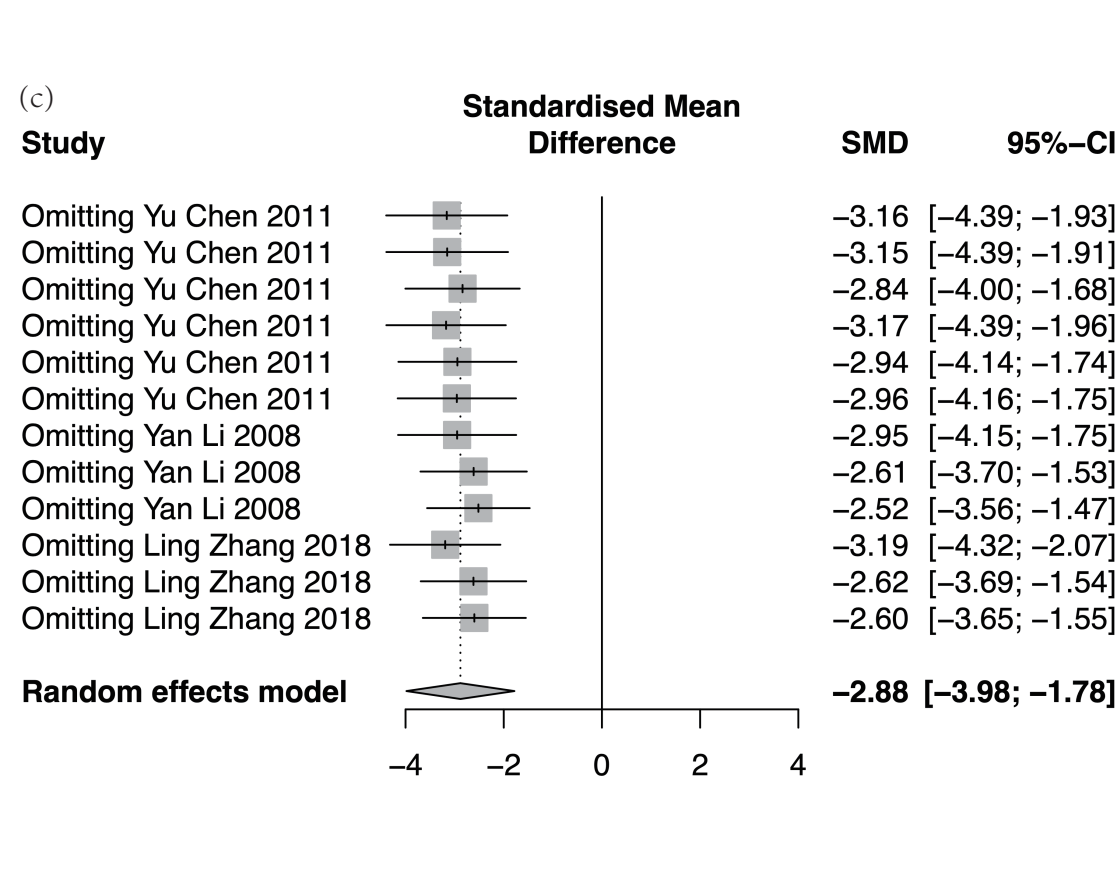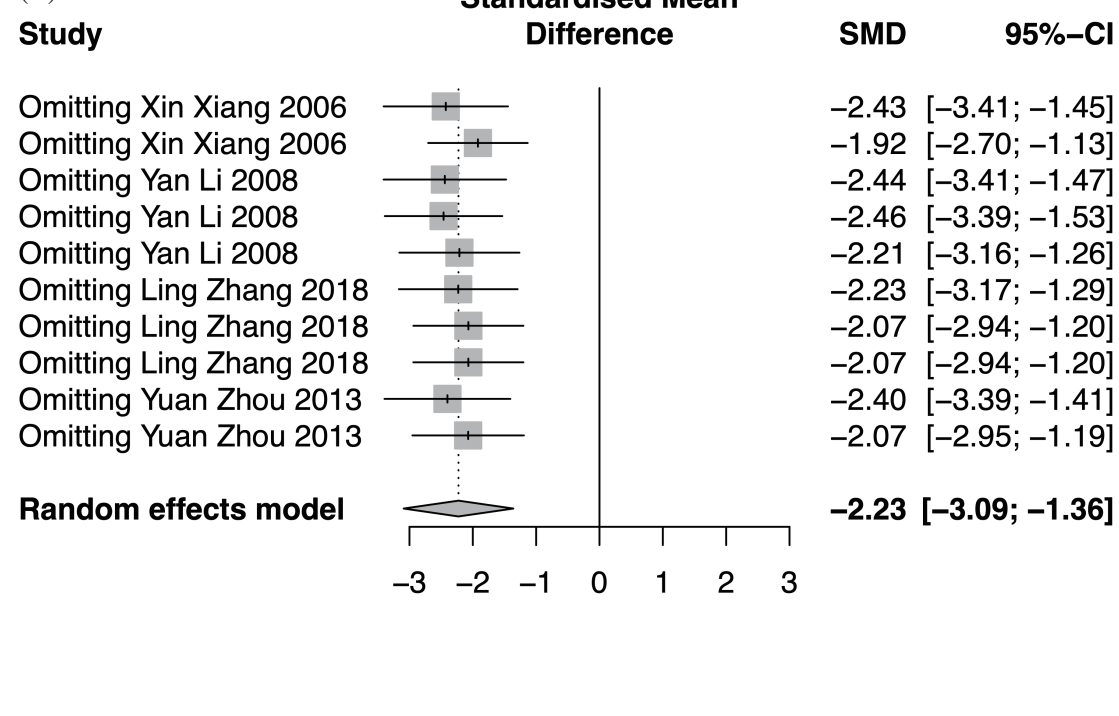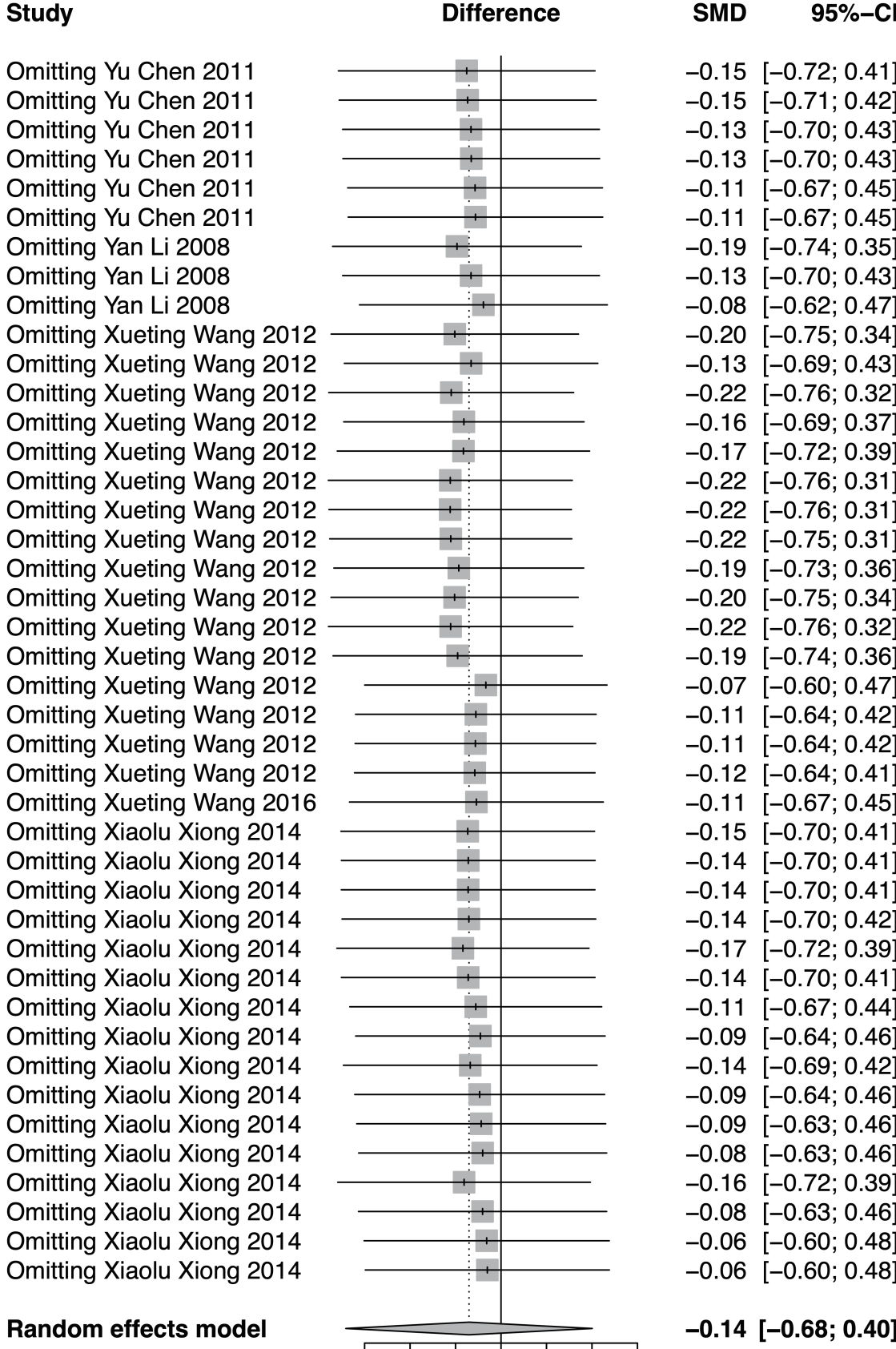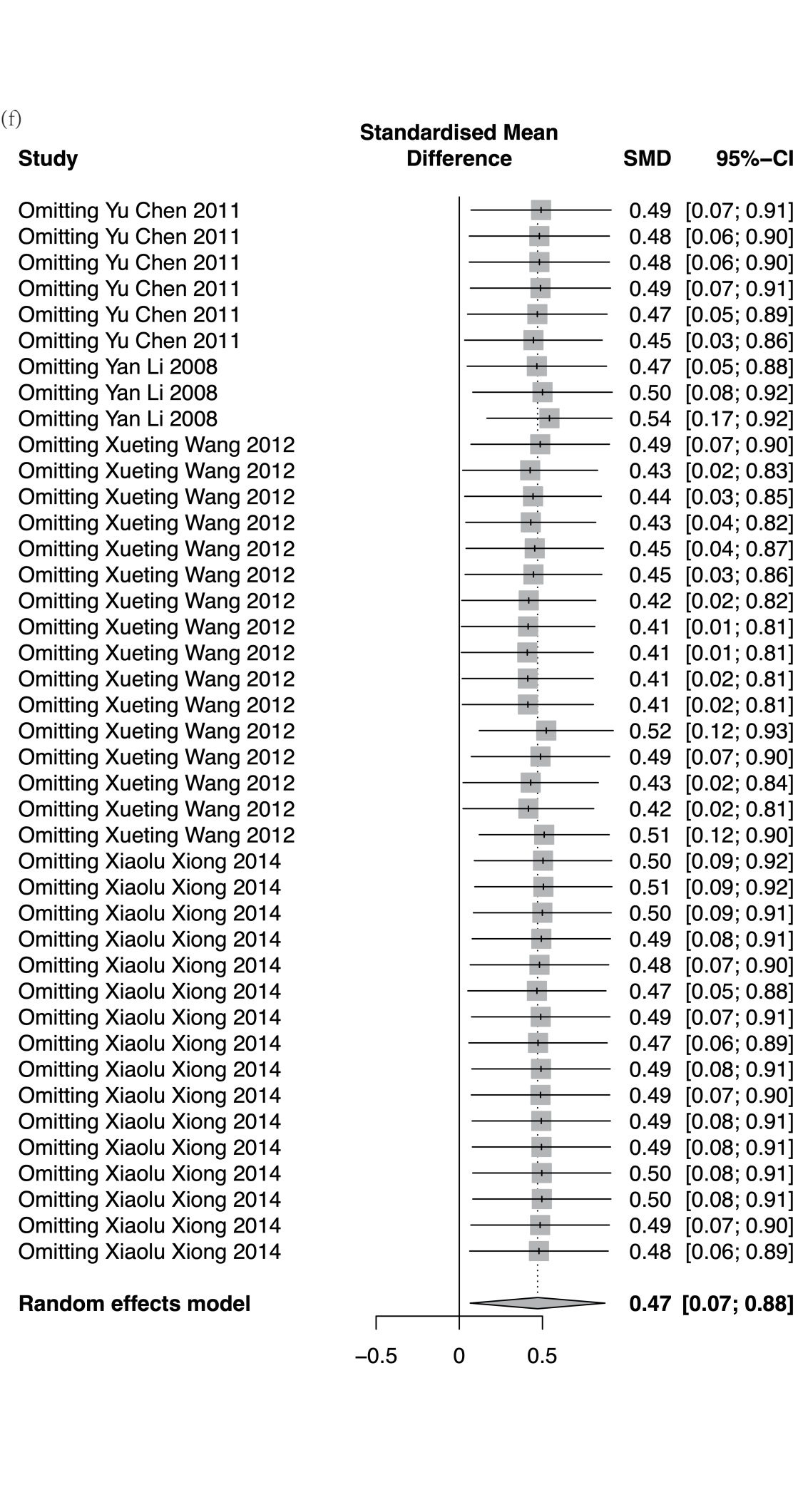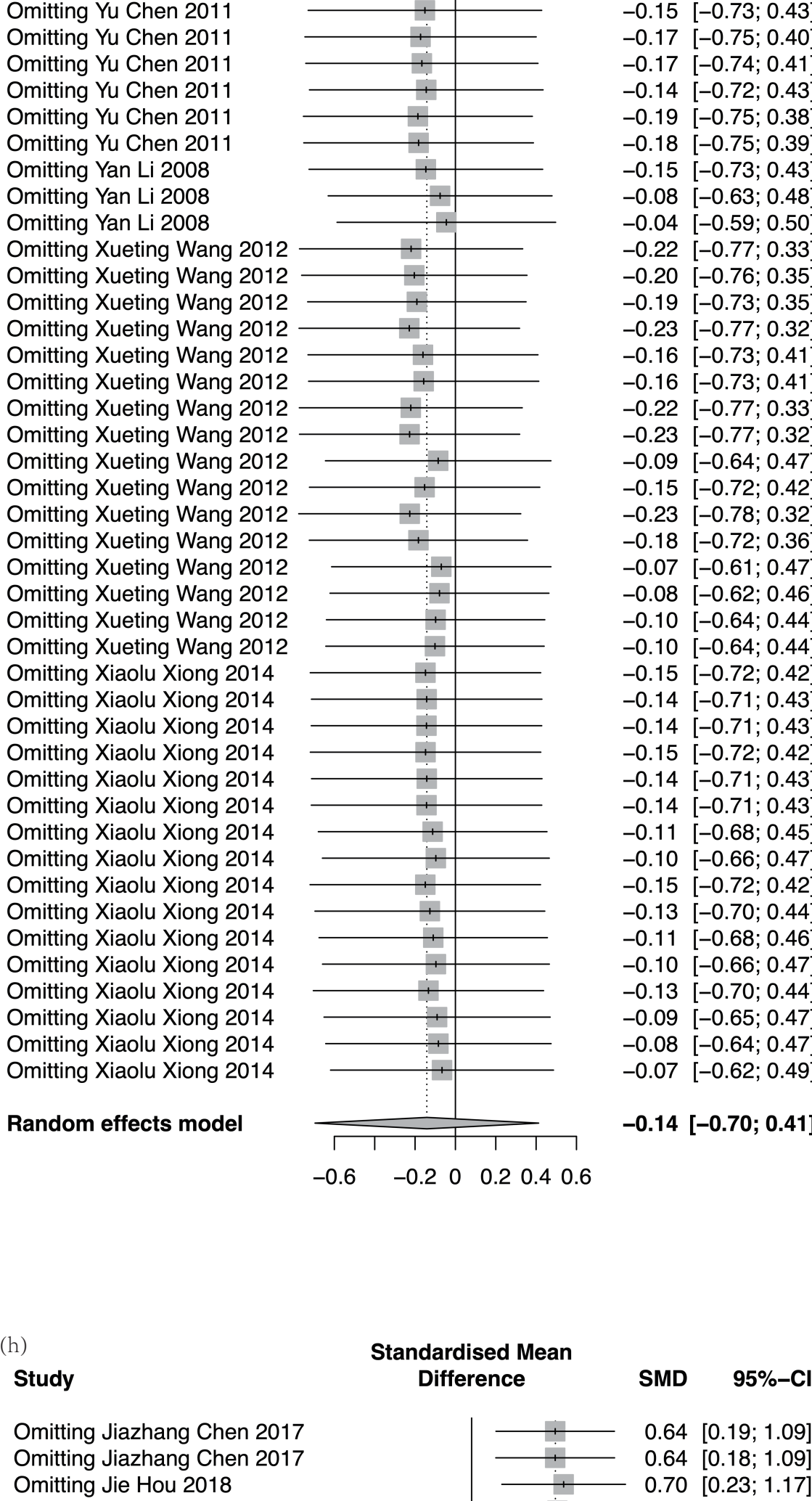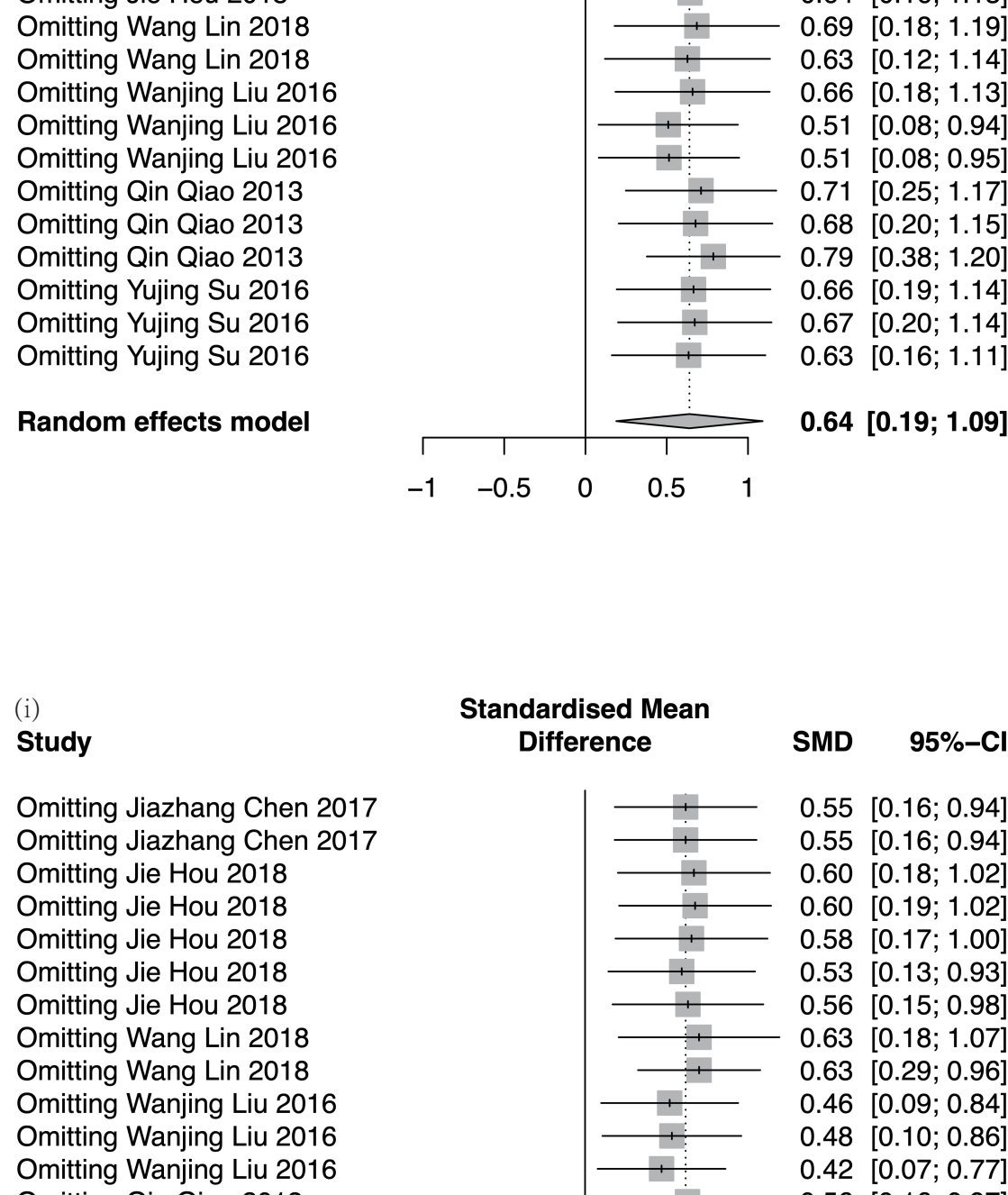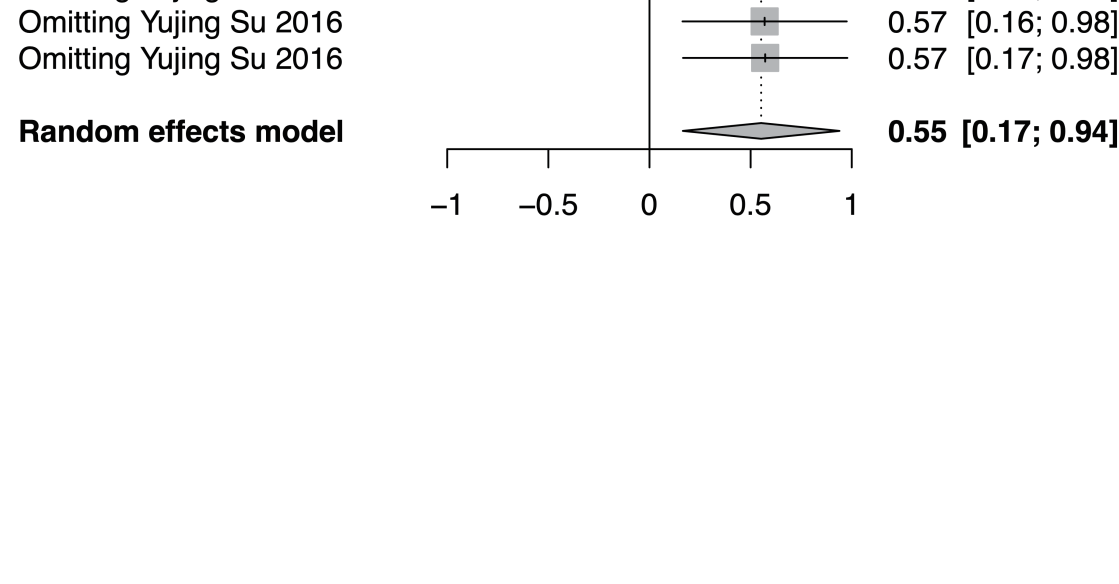

Supplement: Supplementary file 1 [file DataSheet_1.zip › Supplementary Figure 3.pdf]

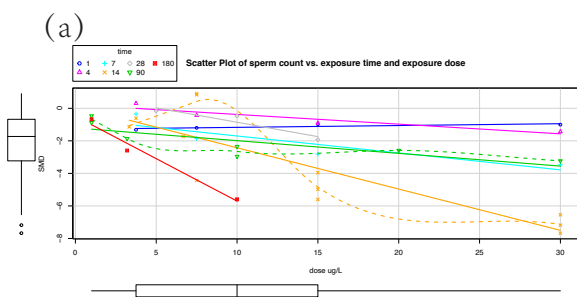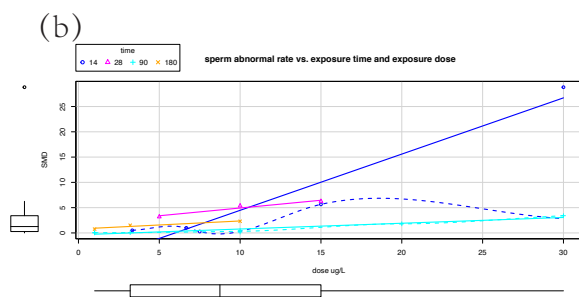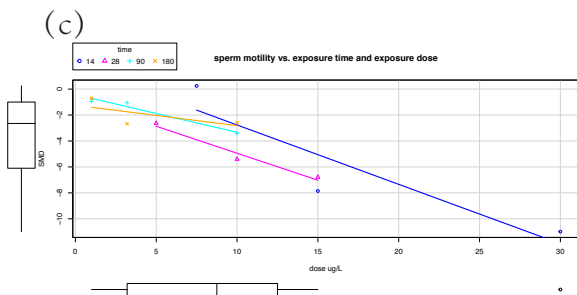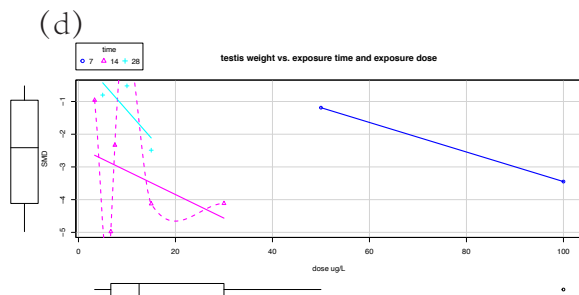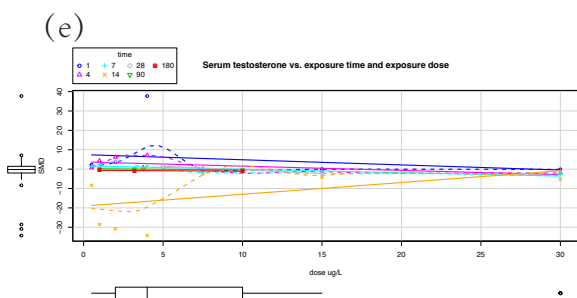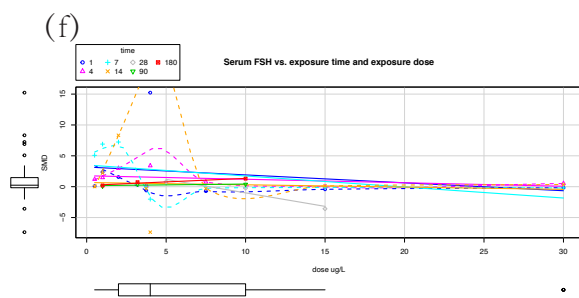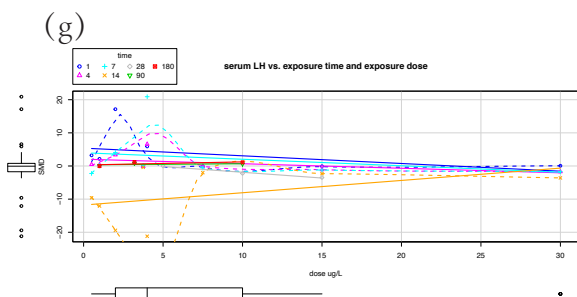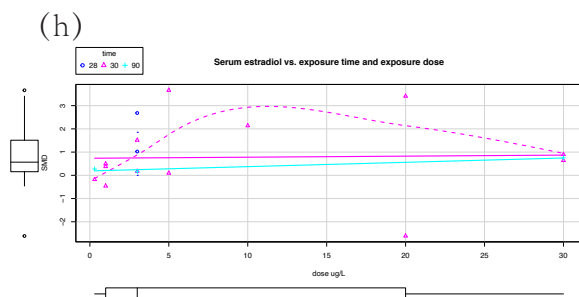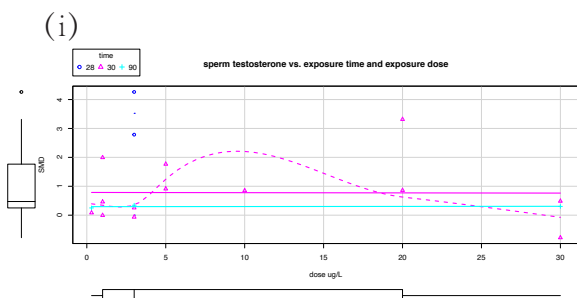

Supplement: Supplementary file 1 [file DataSheet_1.zip › Supplementary Figure 4.pdf]

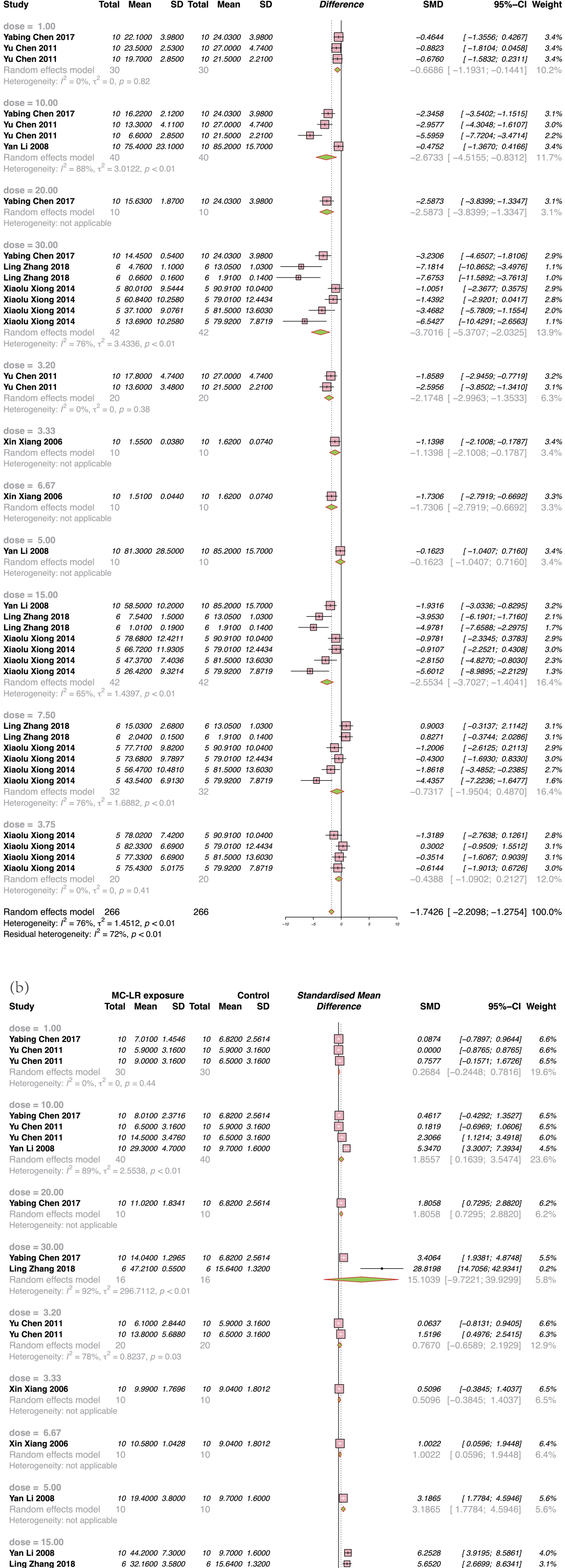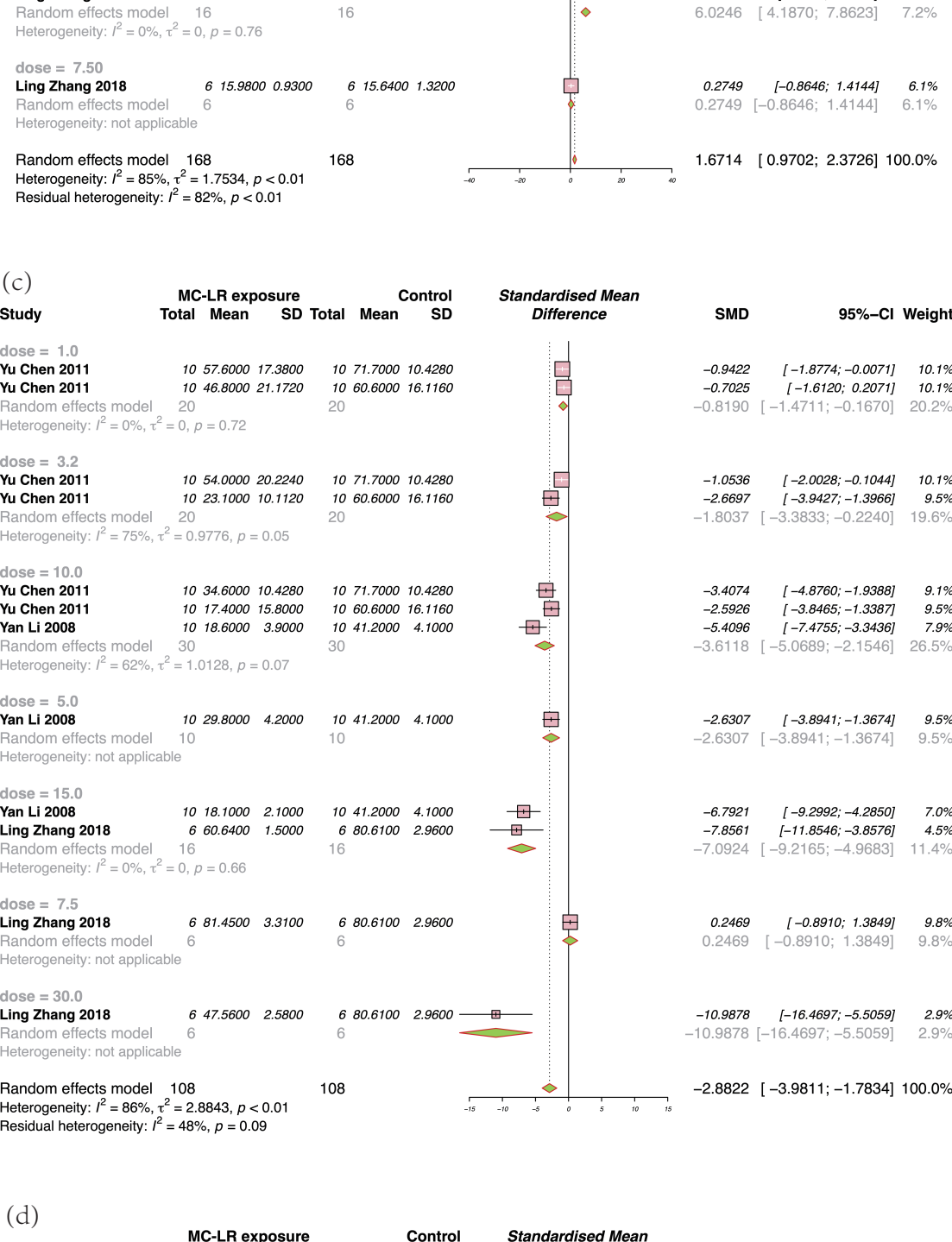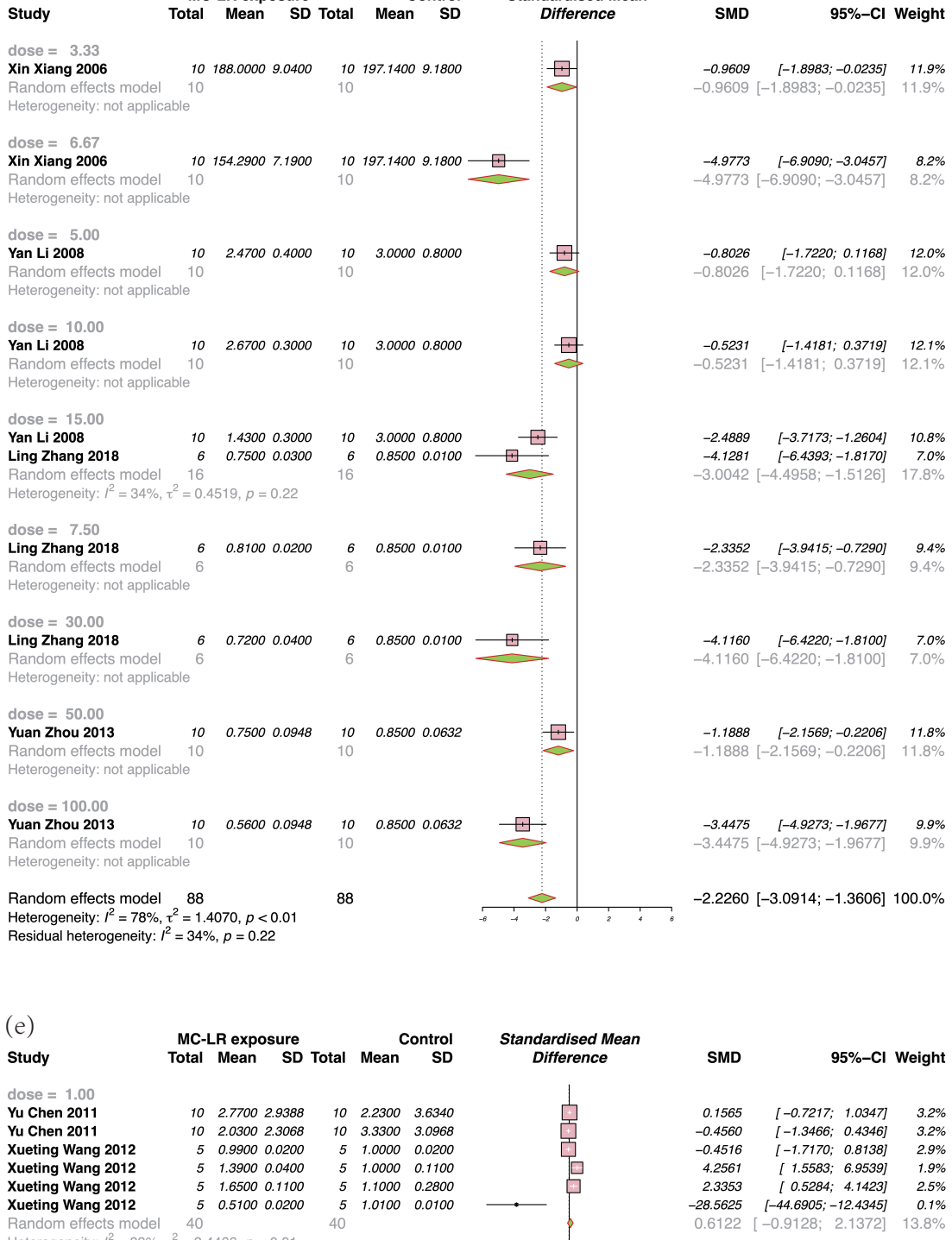

Supplement: Supplementary file 1 [file DataSheet_1.zip › Supplementary Figure 5.pdf]

(a) MC-LR exposure Control Standardised Mean Difference SMD 95%-CI Weight

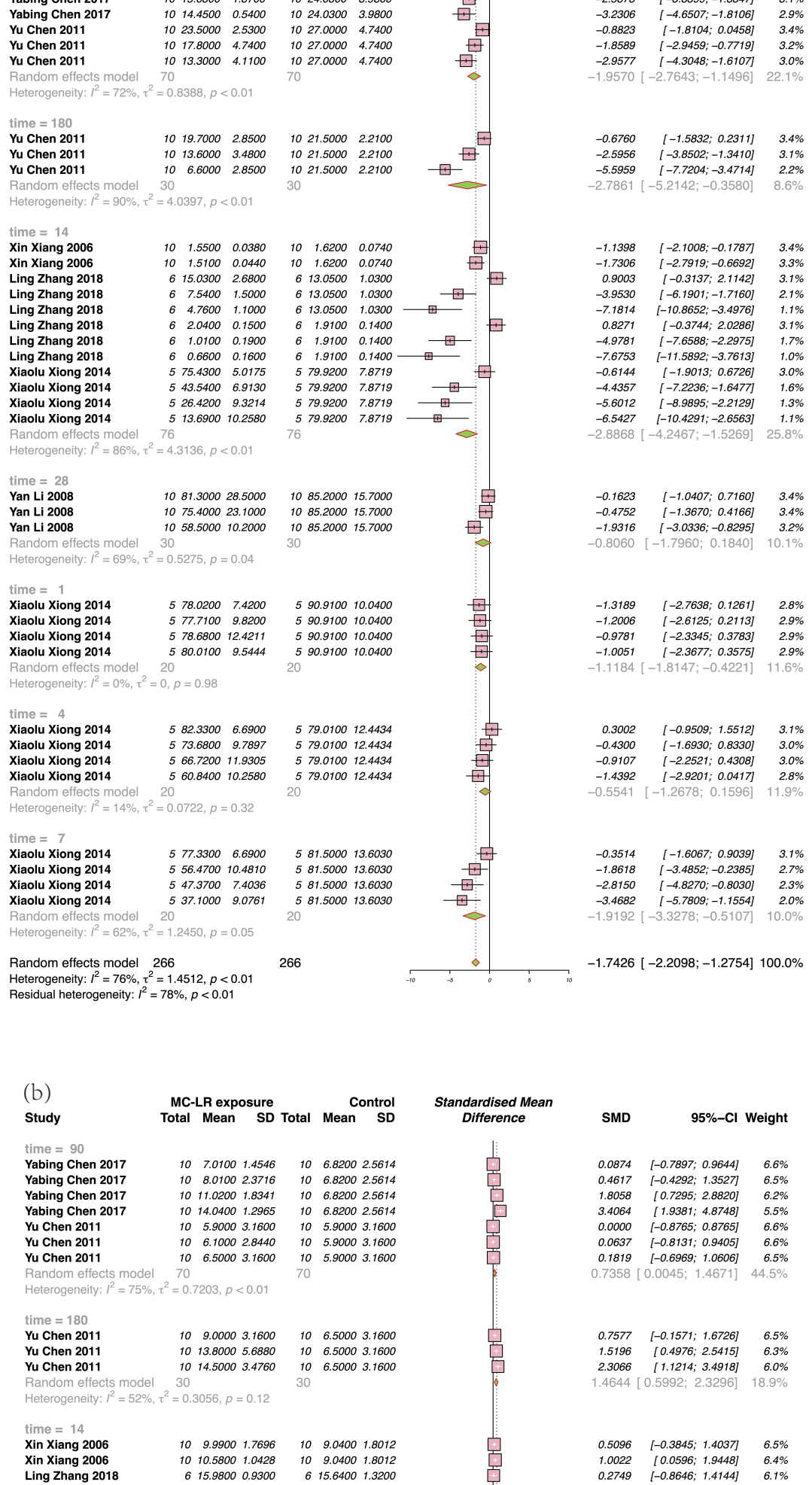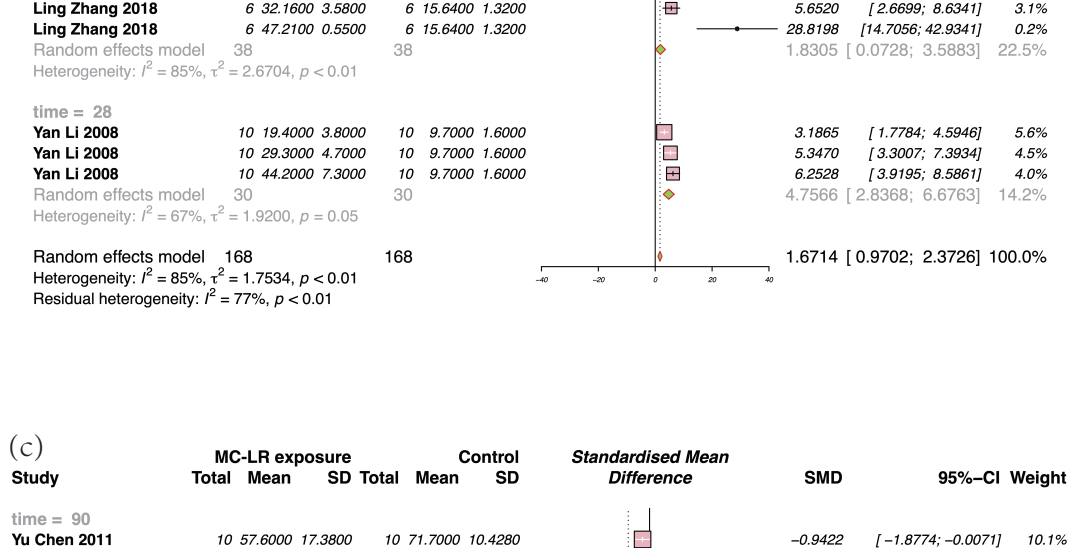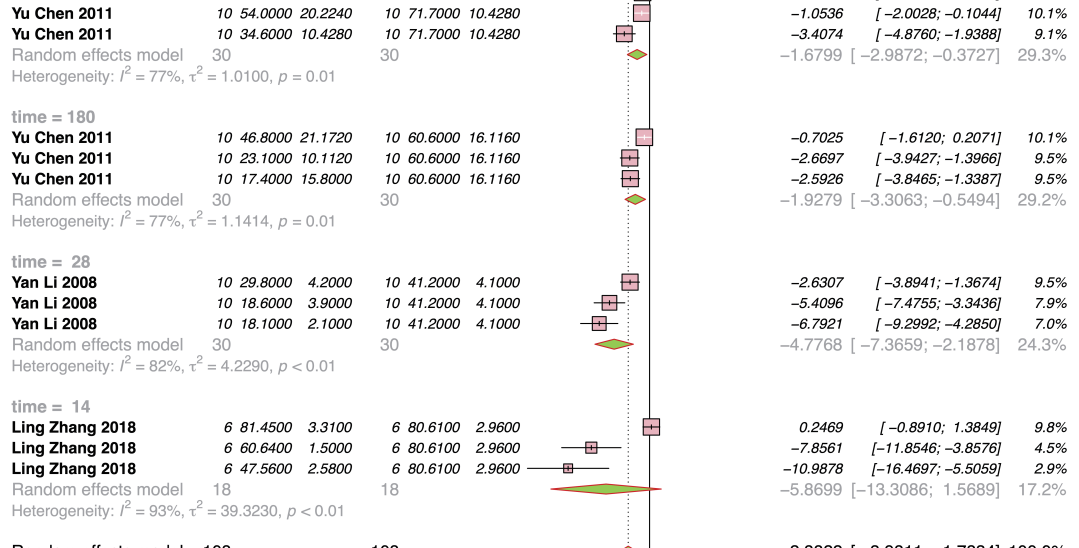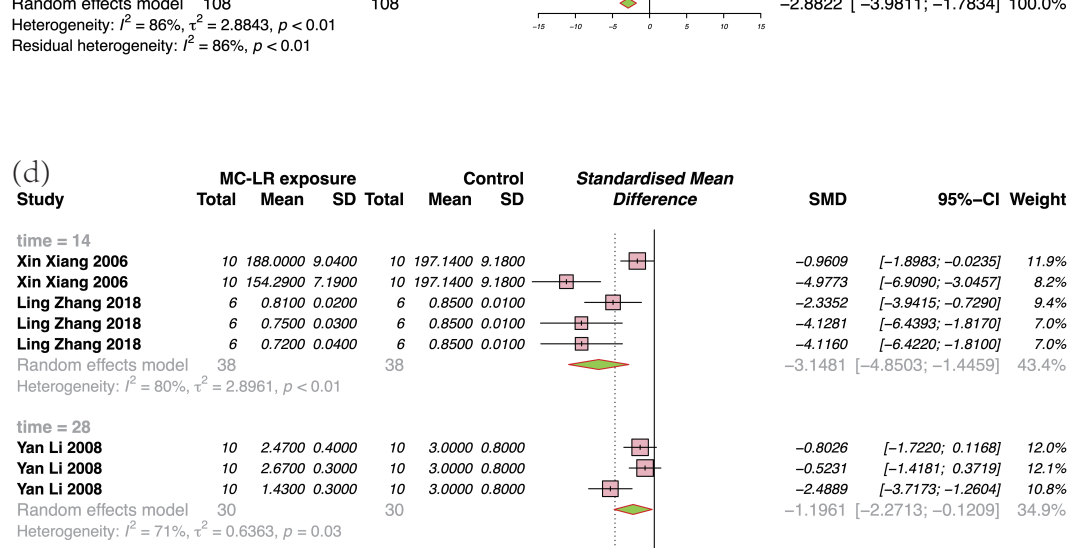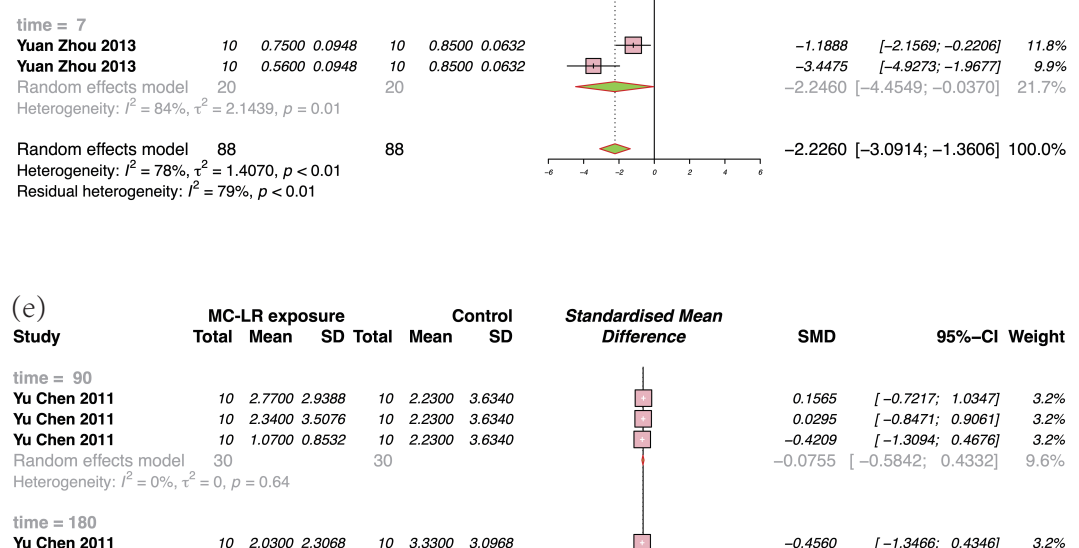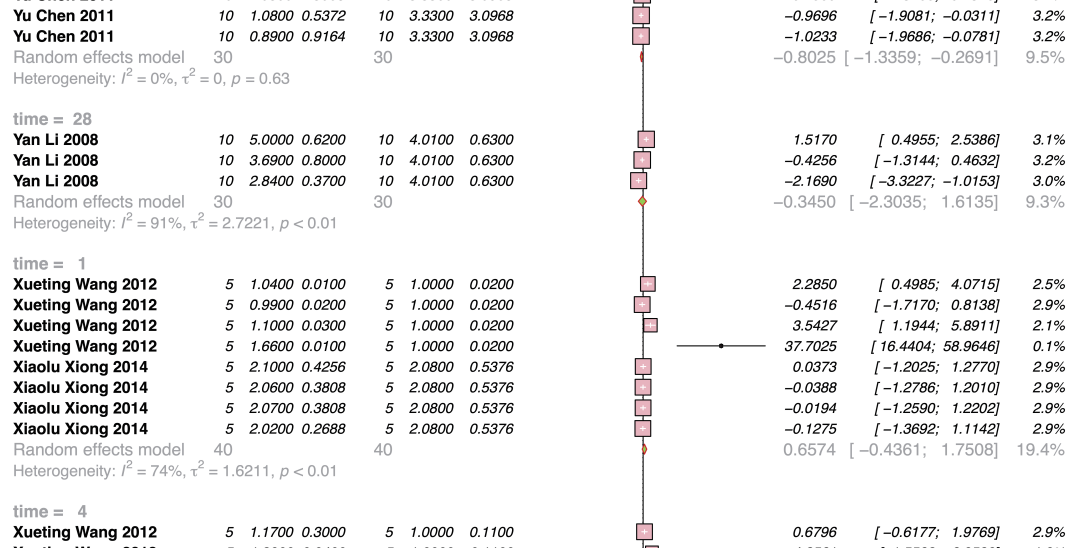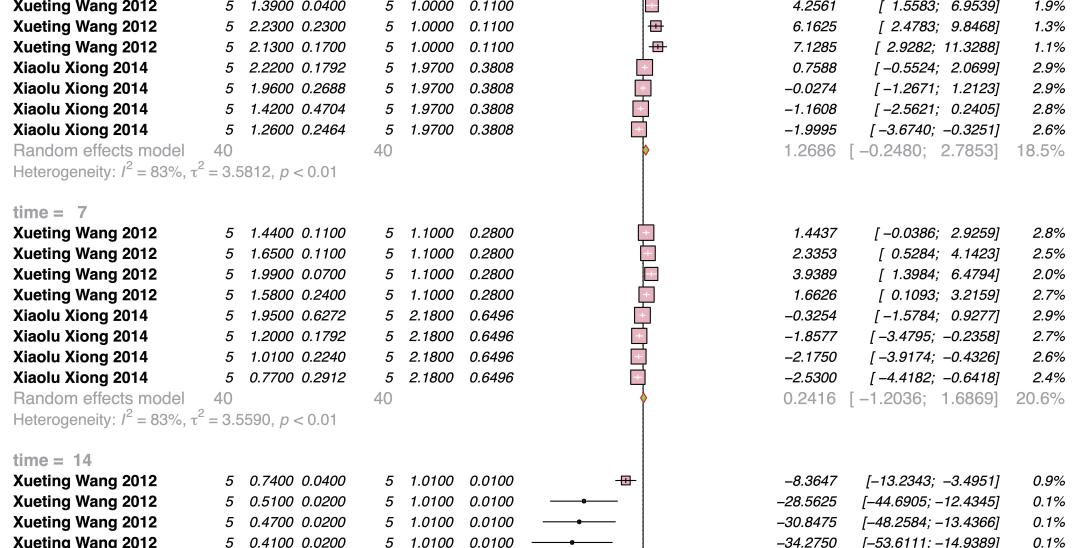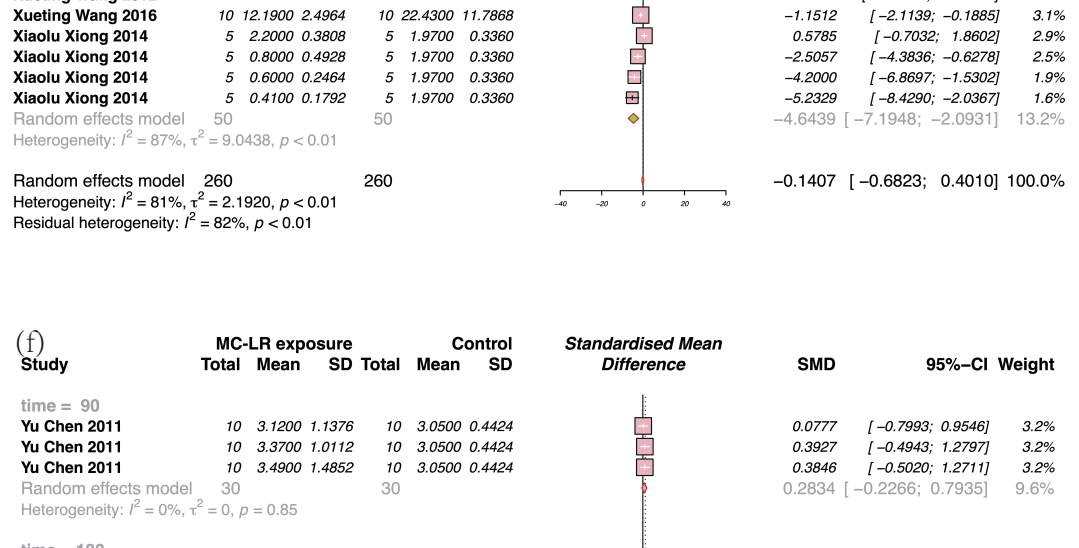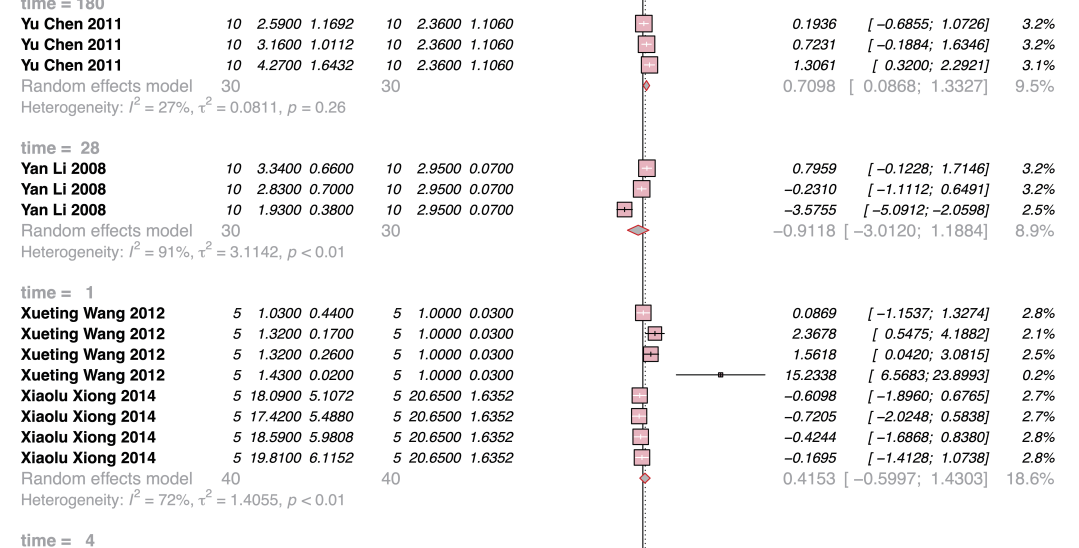

Supplement: Supplementary file 1 [file DataSheet_1.zip › Supplementary Figure 6.pdf]
